# The importance of clinician, patient and researcher collaborations in Alport syndrome

**DOI:** 10.1007/s00467-019-04241-7

**Published:** 2019-05-01

**Authors:** Michelle N. Rheault, Judith Savige, Michael J. Randles, André Weinstock, Melissa Stepney, A Neil Turner, Gina Parziale, Oliver Gross, Frances A Flinter, Jeffrey H Miner, Sharon Lagas, Susie Gear, Rachel Lennon

**Affiliations:** 1 Department of Pediatrics, University of Minnesota Masonic Children’s Hospital, Minneapolis, MN USA; 2 Department of Medicine, Royal Melbourne Hospital, Victoria, Australia; 3 Wellcome Centre for Cell-Matrix Research, Division of Cell Matrix Biology and Regenerative Medicine, School of Biological Sciences, Faculty of Biology Medicine and Health, University of Manchester, Manchester, UK; 4 Alport Syndrome Foundation, Phoenix, AZ USA; 5 Nuffield Department of Primary Care Health Sciences, University of Oxford, Oxford, UK; 6 Renal Medicine, Royal Infirmary, University of Edinburgh, Edinburgh, UK; 7 Clinic of Nephrology and Rheumatology, University Medicine Goettingen, Goettingen, Germany; 8 Department of Clinical Genetics, Guy’s & St Thomas’ NHS Foundation Trust, London, UK; 9 Division of Nephrology, Washington University School of Medicine, St Louis, MO USA; 10 Alport UK, Tetbury, UK; 11 Department of Paediatric Nephrology, Royal Manchester Children’s Hospital, Manchester University Hospitals NHS Foundation Trust, Manchester Academic Health Science Centre, Manchester, UK

**Keywords:** Alport syndrome, COL4A3, COL4A4, COL4A5, Type IV collagen, Basement membrane

## Abstract

Alport syndrome is caused by mutations in the genes *COL4A3, COL4A4* or *COL4A5* and is characterised by progressive glomerular disease, sensorineural hearing loss and ocular defects. Occurring in less than 1:5000, Alport syndrome is a rare genetic disorder but still accounts for > 1% of the prevalent population receiving renal replacement therapy. There is also increasing awareness about the risk of chronic kidney disease in individuals with heterozygous mutations in Alport syndrome genes. The mainstay of current therapy is the use of angiotensin-converting enzyme inhibitors and angiotensin receptor blockers, yet potential new therapies are now entering clinical trials. The 2017 International Workshop on Alport Syndrome in Glasgow was a pre-conference workshop ahead of the 50th anniversary meeting of the European Society for Pediatric Nephrology. It focussed on updates in clinical practice, genetics and basic science and also incorporated patient perspectives. More than 80 international experts including clinicians, geneticists, researchers from academia and industry, and patient representatives took part in panel discussions and breakout groups. This report summarises the workshop proceedings and the relevant contemporary literature. It highlights the unique clinician, patient and researcher collaborations achieved by regular engagement between the groups.

## Introduction

The 2017 International Workshop on Alport syndrome took place in Glasgow and was formally designated as a pre-conference meeting prior to the 50th anniversary meeting of the European Society for Paediatric Nephrology (ESPN). Following on from the previous highly successful workshops in Oxford (2014) [1] and Göttingen (2015) [2], the meeting brought together patients, researchers, clinicians and industry participants from across the world. This review of the workshop proceedings and the contemporary literature highlights the key updates in the following dominant themes that were covered in depth during the 3-day workshop: clinical science and registries, genetics, basic science and the patient perspective.

## Clinical science and registries

**Fig. 1:**
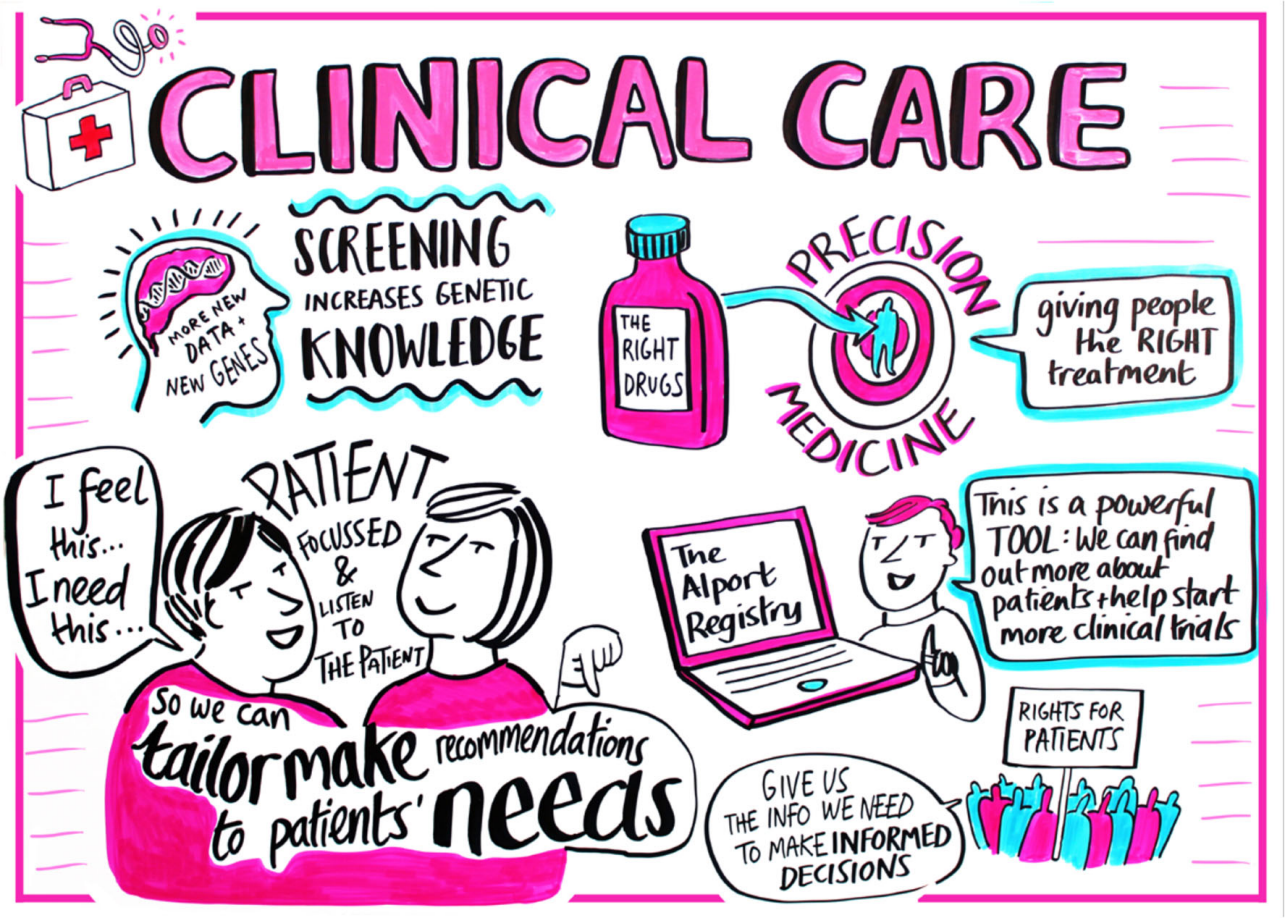
Clinical care in Alport syndrome. The presentations covered patient registries, precision medicine, genetic screening and clinical trials

A large portion of the workshop was focussed on the clinical care of patients with Alport syndrome including care at specific critical junctions such as diagnosis, transition to adult care and pregnancy (Fig. 1). Dr. Rachel Lennon reviewed the diagnostic evaluation of children with persistent haematuria including the importance of obtaining a thorough family history. She also discussed the importance of recognising the diversity of Alport syndrome phenotypes and the role of genetic testing in diagnosis to avoid the need for an invasive kidney biopsy. Individuals presenting with early severe disease may have mutations in additional genes affecting the glomerulus such as *MYO1E* [3]. Dr. Lennon also reviewed the increasingly recognised phenotype of nephrotic syndrome with focal segmental glomerulosclerosis on kidney biopsy caused by mutations in type IV collagen genes [4].

Once a diagnosis of Alport syndrome is made, the next step is determining a plan for follow-up and treatment. These topics were reviewed by Drs. Clifford Kashtan and Oliver Gross. Screening for non-renal manifestations of Alport syndrome is important. Audiology evaluations are recommended once males with X-linked Alport syndrome (XLAS) or males and females with autosomal recessive Alport syndrome (ARAS) are 5–6 years of age. Anyone with type IV collagen mutations and overt proteinuria or clinical concern for hearing loss should have formal audiology evaluations. Ophthalmologic manifestations of Alport syndrome generally develop after adolescence, and screening for males with XLAS or males and females with ARAS should begin at age 15–16 years or sooner if symptomatic. Recommendations for the treatment of Alport syndrome were published in 2013, and these remain the standard of care [5]. Based on retrospective registry data, renin-angiotensin aldosterone system (RAAS) blockade is recommended at the onset of proteinuria regardless of genotype. For individuals with severe mutations or family history of early (age < 30 years) end-stage kidney disease (ESKD), then treatment may be considered when patients are persistently microalbuminuric. Dr. Gross’s phase III randomised placebo controlled study of ramipril treatment in children at very early stages of Alport syndrome (isolated haematuria or microalbuminuria) remains ongoing and is expected to report after 2019 [6]. The results of this trial will help to determine if RAAS inhibition should be recommended at even earlier ages to slow the progression of chronic kidney disease.

Alport syndrome is often diagnosed in childhood, necessitating the transition of care from a paediatric to adult nephrologist. Drs. Arvind Nagra and Neil Turner reviewed the successful ‘Ready Steady Go’ transition program in the UK [7]. This program of educational materials and readiness assessments provides a formal way to ensure smooth transition of children with kidney disease, including children with Alport syndrome. Pregnancy is also an important time of life for women with Alport syndrome; however, little is known about outcomes for women with this disorder [8]. Dr. Matt Hall reviewed outcomes for women with chronic kidney disease at the time of pregnancy and discussed improvements in both maternal and foetal outcomes over the past several decades [9]. Importantly, he reviewed the risks of RAAS inhibition in pregnancy as these are commonly utilised drugs in this population and known to be teratogenic.

Despite widespread use of RAAS inhibition, patients with Alport syndrome are still at risk for progression to ESKD, highlighting the need for clinical trials of novel agents in this population. Prior to initiation of clinical trials, a detailed understanding of the natural history and clinical and biomarker risk factors for progression is necessary. A number of Alport syndrome registries are established around the world and provide valuable natural history information. In addition, an international natural history study of patients with Alport syndrome recently completed follow-up (ATHENA study NCT02136862). At the clinical science breakout session, the group reviewed the current status of each of the registries and agreed to further discussion of a recommended common dataset of information to guide new registry formation and facilitate data sharing between registries. In addition, some of the preliminary data from the ATHENA study was reviewed and publication of results is expected in 2019. The first clinical trial specifically in patients with Alport syndrome started enrolling in 2017. This randomised, placebo-controlled trial of the NF-κB inhibitor bardoxolone (NCT03019185) is a landmark for the Alport research and patient communities, and additional clinical trials are in various stages of planning.

The future of Alport syndrome treatment is bright. Dr. Daniel Gale reviewed the complex pathway of bringing a new drug to market including phase 1, 2 and 3 clinical trials; approval and marketing; and cost considerations. Specific challenges for clinical trials in Alport syndrome include the rare nature of the disease for successful recruiting into clinical trials as well as potential high costs of therapy for patients if a drug is approved. Dr. Michelle Rheault reviewed the importance of understanding the molecular pathways of glomerular filtration barrier dysfunction in Alport syndrome including molecular and cellular changes in podocytes and endothelial cells in response to mechanical strain, as well as changes in composition of the glomerular basement membrane (GBM) [10]. A number of agents are in clinical development for non-Alport syndrome indications that may be appropriate for use in this population based on molecular targets of drug action. For example, endothelin-1 appears to be upregulated in Alport glomeruli in response to mechanical strain and may be targeted by endothelin-1 receptor antagonists currently in development [11].

## Genetics

**Fig. 2:**
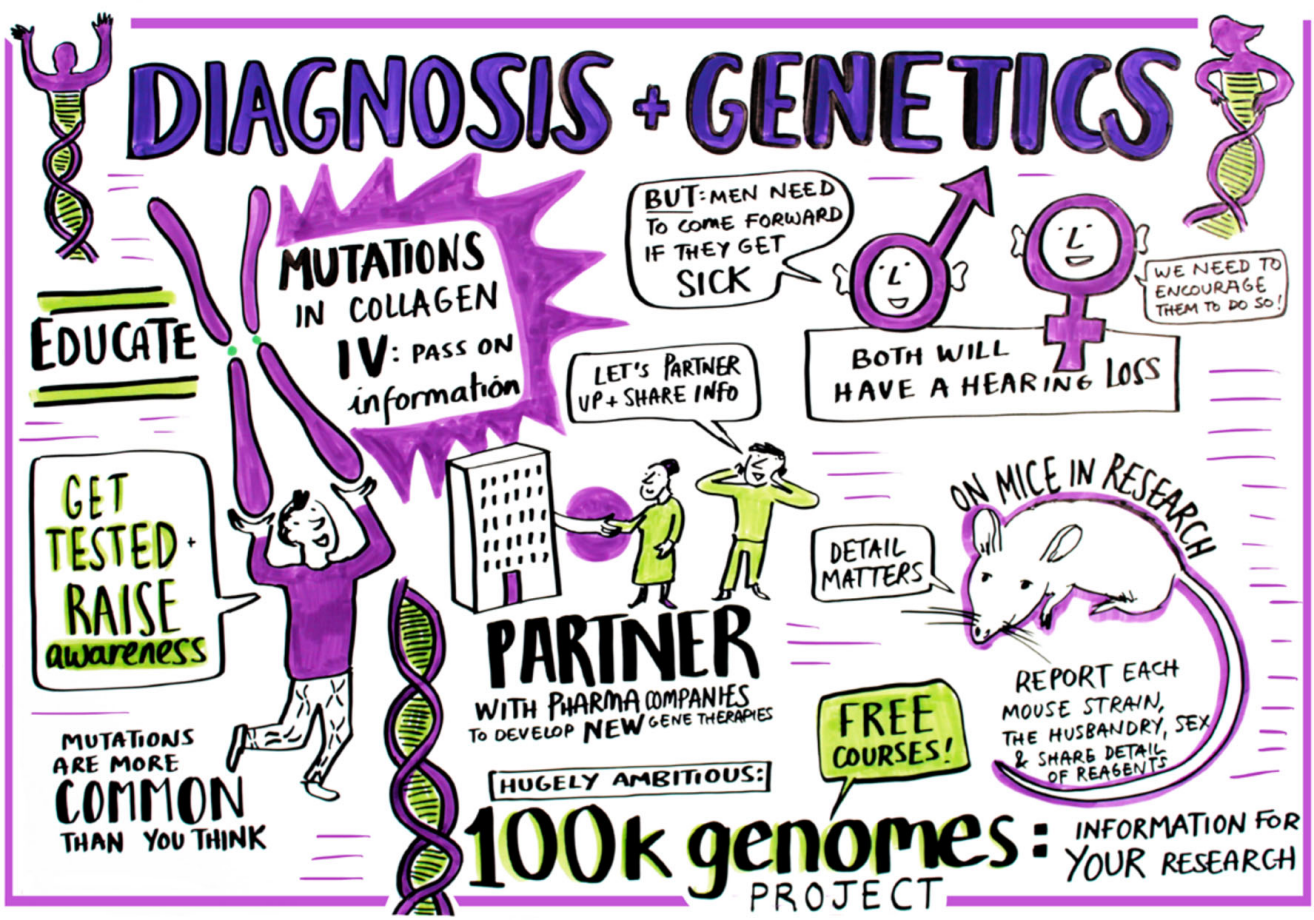
Diagnosis and genetics. The importance of education and raising awareness was discussed together with the classification of Alport syndrome. Genomic sequencing projects such as 100,000 (100 k) genomes in the UK will help inform about the frequency of Alport gene mutations

The presentations on genetics and diagnosis of Alport syndrome demonstrated that our understanding of the value of genetic testing in Alport syndrome has increased greatly over the past 18 months (Fig. 2). In many centres, gene testing has replaced renal biopsy for the diagnosis of Alport syndrome [12]. Identifying the underlying mutation(s) indicates the mode of inheritance, enabling the targeted genetic testing of other family members who may be at risk, as well as sometimes predicting the age at onset of ESKD [13]. Furthermore, some therapies may be specifically targeted at people with missense or nonsense variants in the future.

In addition, *COL4A3*, *COL4A4* or *COL4A5* mutations are found in 10–30% of patients with focal and segmental glomerulosclerosis (FSGS) [4, 14], and Dr. Moin Saleem reported that some patients with features consistent with steroid-resistant nephrotic syndrome had *COL4A* variants on screening with a 70-gene renal panel.

Genetic and non-genetic factors influence the prognosis to a far greater extent than thought previously. Drs. Roser Torra and Alessandra Renieri presented data suggesting that *COL44* mutations result in a more severe phenotype than *COL4A3* mutations. In their experience, up to one third of patients with heterozygous *COL4A4* mutations developed renal impairment at an average age of 56 years. In addition, there is now evidence for testing for coincidental mutations in *NPHS2*, *MYH9* and ACTN4, which all influence proteinuria [3, 15, 16]. In Dr. Constantinos Deltas’ experience, up to 16% of individuals with a heterozygous *COL4A3* or *COL4A4* mutation develop renal impairment and 41% have FSGS. The p.R229Q variant in *NPHS2* predisposes to increased proteinuria and renal failure, and the Rictor gene component of the mTORC2 pathway may be a further modifier of renal function.

For men with XLAS, and men or women with ARAS, the genetic results strongly predict outcome [17, 18], although the clinical course may be modified significantly by screening and early ACE inhibitor treatment [19, 20]. The debate about an appropriate nomenclature for individuals with a heterozygous *COL4A3* or *COL4A4* mutation or a woman with a *COL4A5* mutation continues [21]. For women with a heterozygous *COL4A5* mutation, or anyone with a heterozygous *COL4A3* or *COL4A4* pathogenic variant, our ability to predict the future is more constrained, but these individuals should all undergo life-long renal surveillance [22]. Published data on the risk of ESKD are skewed by ascertainment bias, with an over-representation of people with ESKD in hospital-based series. The risk of ESKD is probably lower in women with a *COL4A5* mutation than historical data suggest, but still higher than in individuals with a pathogenic heterozygous *COL4A3* or *COL4A4* variant.

Some congress participants subscribed to the view that anyone with a heterozygous *COL4A3* or *COL4A4* mutation should be diagnosed with Alport syndrome, to ensure that the significance of their diagnosis is appreciated and to ensure aggressive management and long-term monitoring [21]. Others, while agreeing on the need for life-long follow-up, do not classify people with a single mutation as having Alport syndrome, because of their lesser risk of ESKD and absence of extra-renal manifestations. If every person with a heterozygous *COL4A3* or *COL4A4* mutation were labelled with the diagnosis of Alport syndrome, its incidence would increase from the current level of 1 in 5–10,000 to as high as 1 in 100 [23, 24], with ESKD being uncommon. At the meeting, there was, however, unanimous agreement about the need for long-term monitoring, screening of other family members and a low threshold for prescribing ACE inhibitors to treat proteinuria or hypertension. Clearly, more comprehensive genotype/phenotype data are needed to enable an objective calculation of the risk of renal impairment in heterozygous carriers. Single mutations are significant susceptibility factors for renal impairment in later life, but other factors, including lifestyle attributes such as obesity, diabetes, exercise, untreated hypertension and proteinuria, are important too. Pregnancy-associated hypertension represents a risk factor in women with heterozygous *COL4A5* mutations, and close monitoring is required throughout each pregnancy.

Whole-exome and whole-genome studies are identifying many more genetic variants in Alport genes, but this abundance adds to the work of confirming pathogenicity. The American College of Medical Genetics Guidelines for the classification of variants are very clear [25], and their universal adoption, together with submission of variants to the Leiden Open Variation Database (LOVD) or other databases, will help us understand the significance of individual variants better, especially heterozygous variants in the *COL4A* genes.

## Basic science

**Fig. 3:**
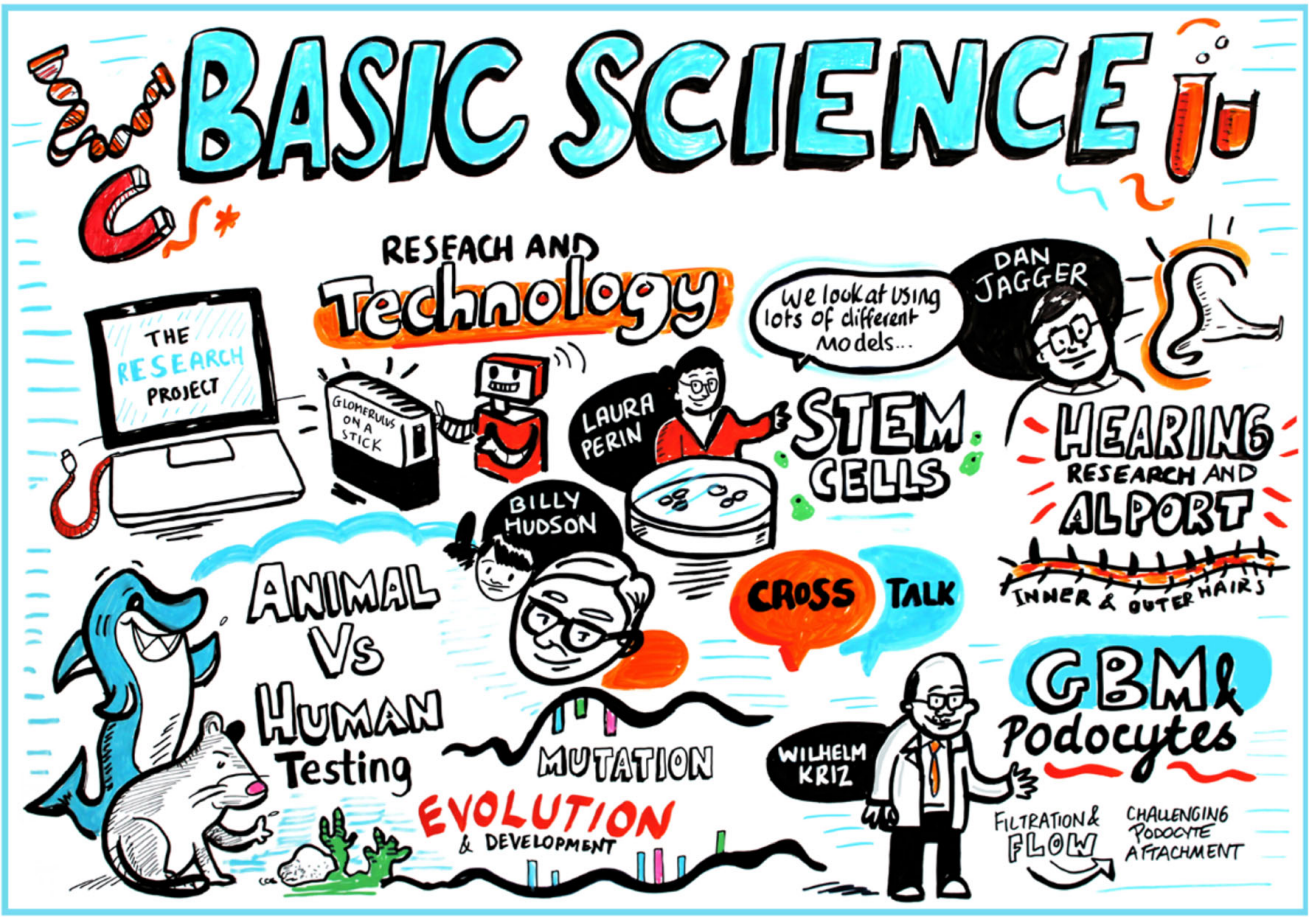
Basic science. The presentation covered the different experimental systems in use to investigate the biology of type IV collagen and basement membranes. There was a focus on the mechanisms of hearing loss in Alport syndrome

As with previous Alport workshops, there were exceptional speakers who delivered talks regarding fundamental research relevant to Alport syndrome (Fig. 3). Dr. Billy Hudson, from Vanderbilt Medical Centre, gave an impassioned talk where he emphasised the critical importance of understanding the basic molecular mechanisms governing the ‘molecular rope’ formed by the three type IV collagen alpha chains that, when defective, cause Alport syndrome. His research group recently investigated type IV collagen at the evolutionary dawn of metazoan tissues by utilising Ctenophora. This is due to Ctenophora being one of the earliest branching extant animal phyla. Their studies revealed that type IV collagen is present in Ctenophora but absent in unicellular sister-groups, thus suggesting that type IV collagen is one of the fundamental architectural units for multicellular tissue genesis [26]. Dr. Hudson stressed the key role of protein–protein interactions, not only intra-type IV collagen interactions but also of the interactions of type IV collagen with other basement membrane (BM) components, including laminins and nidogens, in addition to cell surface receptors such as integrins.

Dr. Wilhelm Kriz followed up Billy Hudson’s talk by first presenting data on the mesangium as a ‘dumping ground’ for defective and degraded glomerular BM (GBM) material. Evidence from the 1970s suggests that the GBM is turned over approximately every 65–100 days [27], and undoubtedly, this turnover is essential for a normal functioning GBM. Dr. Kriz demonstrated, using transmission electron microscopy (TEM), in addition to staining for specific GBM markers, that the mesangial matrix contains turned over GBM material. Furthermore, he showed evidence that increased production and defective degradation of GBM during diabetic nephropathy (DN) contributes towards mesangial matrix expansion [28]. He then drew parallels between DN and Alport syndrome by analysing Alport patient TEM samples. Dr. Kriz hypothesised that the defective GBM component in Alport syndrome is likely to be shunted to the mesangial compartment in a mechanism similar to that seen in DN. This process may have an important role in Alport syndrome, leading to expansion of the mesangial matrix compartment and eventual loss of glomerular function. Interestingly, Dr. Kriz went on to explore the role of mechanical force within glomeruli and the consequent impact on Alport pathology. The glomerular capillary wall is exposed to high flow rates causing sheer stress [29]. The GBM is known to create the main wall tension during glomerular filtration; however, this causes the GBM to expand, and at a critical point, the GBM can no longer cope with the forces involved. Therefore, contraction of podocytes [30, 31] and mesangial cells [32] is required to contribute to withstand the forces across the glomerular capillary wall. Dr. Kriz described how the location of podocytes, attached to the outside of the GBM, leaves them susceptible to detachment and loss from the glomerulus in the urine. Moreover, podocytes located close to the hilum are more susceptible to this process because of the high flow rates; it is likely that podocytes that detach here cause a local increase in force that prevents reattachment [29].

**Fig. 4:**
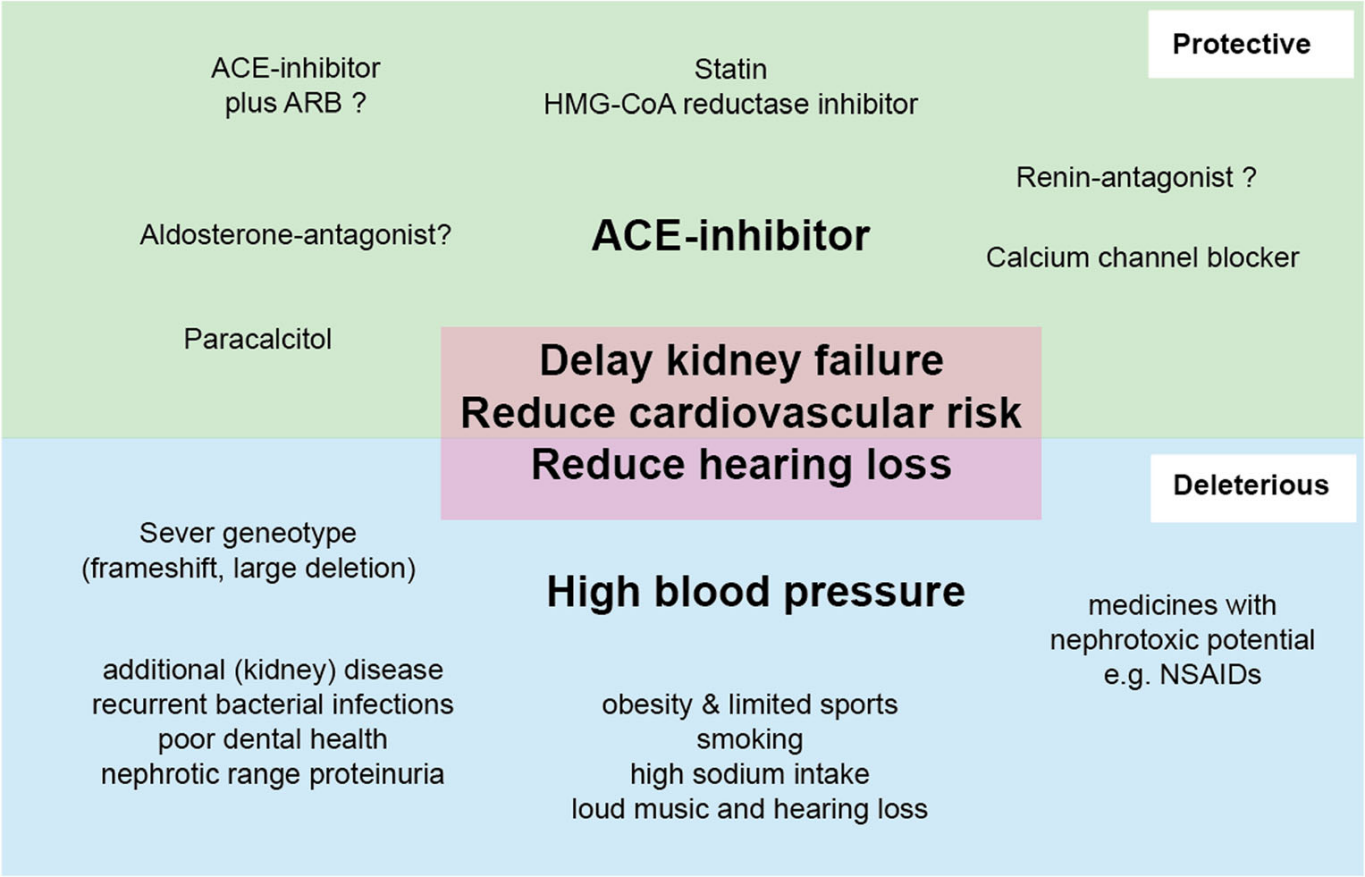
Factors that influence the progression of Alport syndrome. Treatments that have confirmed or proposed effects on protecting kidney function, cardiovascular risk and hearing (top half in green) and factors that are known or thought to be deleterious (bottom half in blue). ACE angiotensin-converting enzyme, ARB angiotensin receptor blocker, HMG-CoA β-hydroxy β-methylglutaryl-CoA, NSAIDs non-steroidal anti-inflammatory drugs

Dr. Oliver Gross presented work from his laboratory exploring the role of environmental modifiers of glomerular disease such as obesity, high calorie intake and high sodium, in addition to treatment with angiotensin-converting enzyme inhibitors, the most common treatment for Alport patients (Fig. 4). Mouse models of both steroid-resistant nephrotic syndrome and Alport syndrome are being used, and this approach has the potential to discover the efficacy of the standard treatment for Alport syndrome taking into account many environmental factors.

Mouse models and human patient data are the most frequently used in the Alport research community, which has led to major discoveries in the past 30 years. However, both higher throughput systems including zebrafish, which have utility for high throughput screens and high-resolution in vivo imaging, and larger mammalian models of Alport syndrome are required to bridge the gap in Alport research. Dr. Mary Nabity is studying a canine model of XLAS. These animals have a lifespan of around 1 year, and Dr. Nabity has analysed RNA-seq samples collected at clinical milestones of glomerular disease development in these animals [33]. From this study, Dr. Nabity has discovered new genes that are differentially expressed between the slow and rapid progressing animals with Alport syndrome, including lysyl oxidase (LOX) transcripts. Further studies into the role of these transcript changes may provide powerful insight into the mechanisms leading to slow or rapidly progressing Alport syndrome. Another key area of research pertinent to Alport syndrome is regenerative medicine. In recent years, there has been tremendous progress in the generation of protocols for the differentiation of stem cells into renal progenitors and kidney organoids [34, 35]. Dr. Laura Perin is seeking to apply regenerative medicine to derive a treatment for Alport syndrome. She has developed systems involving stem cells taken from amniotic fluid [36] and has discovered a potential role for extracellular vesicles, produced by stem cells, in modulating vascular endothelial growth factor (VEGF) signalling in glomerular pathobiology. Deeper understanding of this process may indeed lead to potential drug targets in Alport syndrome.

Protocols for isolating and culturing podocytes from urine have been employed in a number of studies [37], and although known to be challenging, these approaches enable researchers to gain insights into podocyte biology. Dr. Sergio Daga, from The University of Siena, has been using this approach to isolate podocytes and subsequently use CRISPR gene editing technology to study Alport syndrome. This area of research has the potential to unlock further mechanistic insights in Alport syndrome.

The therapeutic potential of anti-miR-21 for the treatment of Alport syndrome was first investigated by Dr. Jeremy Duffield and colleagues in 2015 with promising results [38]. Dr. Tsubasa Yokota from Dr. Hirofumi Kai’s laboratory has been building on some of these findings from this study. STAT3 and IL6 were found to be increased in mice with Alport syndrome before anti-miR-21 treatment; the group, therefore, investigated the impact of STAT3 and IL6 inhibitors in Alport mice. They found that STAT3 inhibition reduced proteinuria, whereas IL6 inhibition had no effect on this metric of glomerular function [39]. This is not the only treatment that the group have tested in Alport mice; bromide supplementation was also tried, as Billy Hudson had shown bromide to be important for type IV collagen network formation [40]. However, bromide supplementation exacerbated Alport pathology [41]. Dr. Kai’s research group investigations into both the repurposing of licensed drugs, including metformin, and novel therapies are of vital importance for the Alport community.

Despite renal pathologies being the focus of the Alport workshop, there is a desperate need for more research into the hearing impairment caused by Alport syndrome. Dr. Dan Jagger, from University College London, delivered a talk to provide some insights into the sensorineural hearing loss found in individuals with Alport syndrome. It is known that hearing loss occurs in Alport syndrome, but there is no evidence for a disorder in balance, which may be anticipated if there is progressive damage to the inner ear. The structure thought to be most likely progressively damaged in Alport patients is the basilar membrane of the cochlea. The basilar membrane sits on a basement membrane which is composed of type IV collagen a3a4a5. Both inner and outer hair cells of the cochlea are exposed to forces, which in turn are converted to electrical signals interpreted by the brain as sound. Evidence suggests that it is the outer hair cells that are damaged in Alport syndrome. The outer hair cells are the ‘amplifiers’ of the signal generated by the inner hair cells, which is why a ‘base level’ of hearing that can be amplified with hearing aids remains in individuals with Alport syndrome. Although it is uncertain whether the hearing loss phenotype is present in all mouse models with Alport syndrome, Dr. Jagger warned about the future of hearing research in Alport mice, as it is well known that there is age-related hearing loss in mice, which may confound studies. The most obvious current advice to patients with Alport syndrome is to avoid exposure to mechanical damage, for instance use of head phones.

## Patient perspectives

Representatives from National Patient Organizations were in attendance from Australia, China, France, Germany, Italy, The Netherlands, Spain, the UK and the USA. In addition to attending the main session, a pre-event meeting took place with these groups to discuss how they could better collaborate with each other to provide improved resources and support to patients and encourage research. The creation of an International Alport Organization was discussed to achieve this and elements of this organization were captured by the meeting illustrators. It was agreed that there are significant challenges to the creation of such an organization. Amongst these were:

- Dependence on volunteers for organization and resources.
- Significant differences in treatment resources and needs between different countries.
- Differences in effective communication modes.
- Differences in cultural attitudes towards disease, privacy and the physician–patient relationship

These challenges closely mirror those posed to creation of an International Alport Patient Registry discussed in past workshops. Susie Gear of Alport UK proposed a series of potential logos for an International Alport Organization (Fig. 3).

**Fig. 5:**
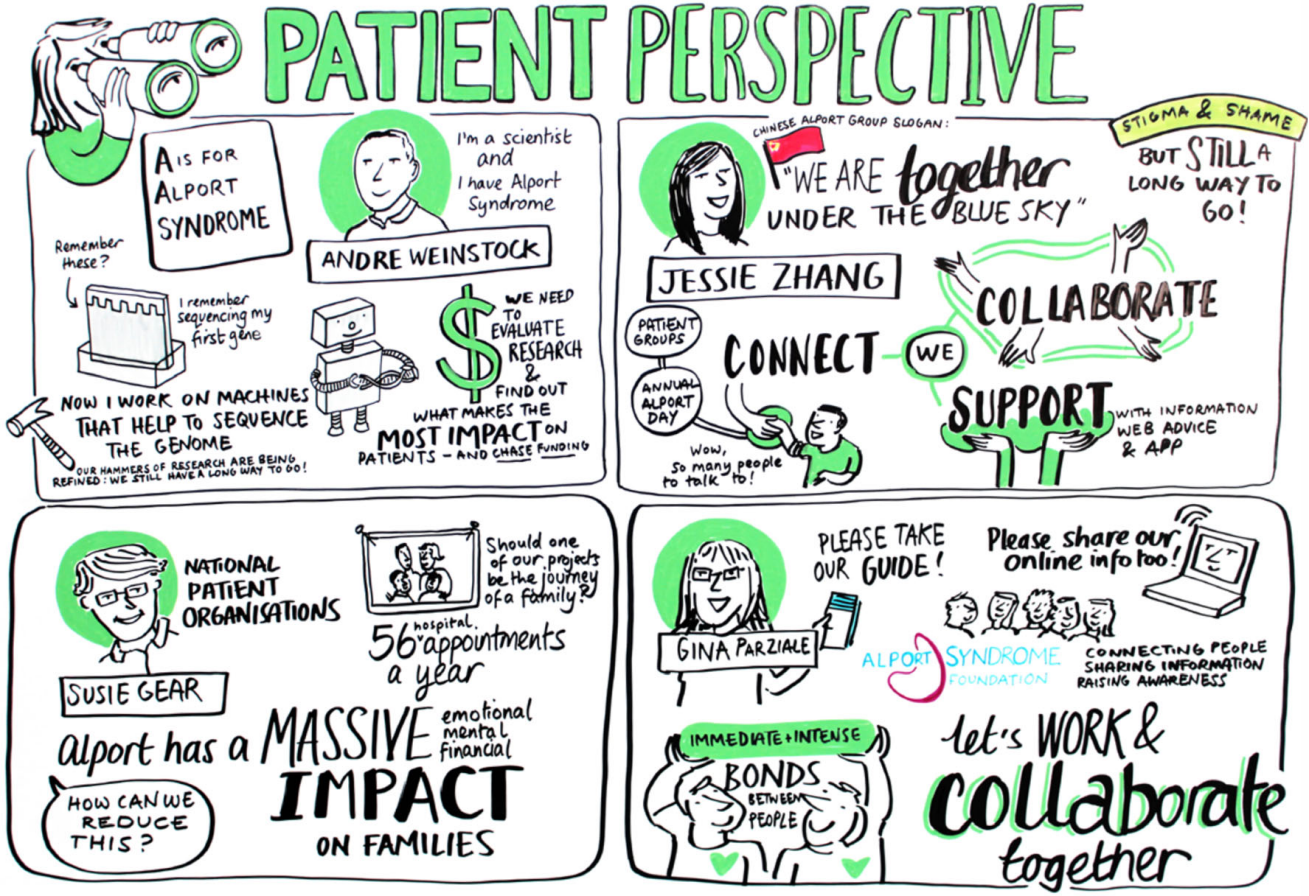
Patient perspective. Four presentations from members of Alport syndrome patient organisations highlighted the importance of the patient voice

Recognising the importance of the patient perspective in research and the development of potential therapies, patients were asked to share their stories with the larger group (Fig. 5). Andre Weinstock from the USA shared his experience as a patient who had a transplant and continues to experience hearing loss. This led to a discussion about the role of hearing loss in Alport syndrome as an important quality of life concern. Jessie Zhang, a mother of an Alport patient from China, spoke about the need for patients to connect with each other as part of a community to alleviate feelings of isolation, gain knowledge and resources, and work together for advocacy. The Alport Collective, a group of teenage patients from the UK, shared videos they created to improve communication among teens about Alport syndrome, and these are accessible on YouTube: https://www.youtube.com/watch?v=Ps3rMpx7YZg, https://www.youtube.com/watch?v=5gJEtmhcClU. Maria Jose, from Spain, spoke of the patient advocacy organization’s efforts to unite their community. While each journey was different, all conveyed the importance of having a community to provide support and resources.

In addition, patient groups play a critical role in research and the development of potential therapies. These groups are instrumental not only in recruiting patients for research but also conveying the patient perspective to researchers and industry. Gina Parziale, Executive Director of the Alport Syndrome Foundation in the USA shared their plans for an Externally Led Patient Development Meeting. It took place in San Diego in October 2018 and brought together patients and their care-partners, representatives from the US Food and Drug Administration (FDA), pharmaceutical companies interested in developing drugs for the disease, and physicians—all to hear from patients about Alport syndrome. In these meetings, the patient’s experience is brought to the forefront for governmental regulatory bodies, pharmaceutical companies and academic researchers to understand.

## The Healthtalk project on Alport syndrome

As a further expansion of the patient voice, the meeting also heard about the Healthtalk project on Alport syndrome. The importance of listening to patient experiences is central to formulating health policy in the UK (and many other countries) [42]. Research suggests that hearing other people’s experiences, alongside factual information, engages people’s attention and influences and supports treatment decision making [43, 44].

From 2015 to 2017, a research project collaboration between Alport UK and the University of Oxford led to a freely accessible resource on Alport syndrome at the website Healthtalk.org. Based on rigorous qualitative research, Healthtalk provides a multi-media internet resource on the experiences, information and support needs of people with different health issues. The site already features 110 different conditions, had more than 6 million visitors in 2018 and is based on more than 4,000 in-depthnarrative interviews of peoples’ experiences of health and illness. Health conditions on the site include several major cancers,cardiovascular disease, mental health, epilepsy, rheumatoid arthritis, pregnancy, screening, sexual health and experiences of carers ofpeople with dementia. The interviews are generated and analysed by experienced social science researchers, most of whom are based in the Health Experiences Research Group within the University of Oxford’s Nuffield Department of Primary Care Health Sciences www.phc.ox.ac.uk/research/health-experiences. Dr. Melissa Stepney (co-author) conducted the interviews and analysis for the section on Alport Syndrome. The Healthtalk sections have direct relevance for health and social care professionals and students, schools, undergraduate and post-graduate learning and teaching.

Further, the website section on Alport syndrome provides an evidence base for patients and the public, learning and teaching, policy makers, all who want to consult a balanced, patient-led collection of experiences. Here, narrative interviews were conducted with 38 people with Alport syndrome, including families and partners, and 3 interviews with healthcare professionals with expertise on Alport syndrome. The patient interviews offer in-depth knowledge and first-hand experiences of what it is like to actually live with Alport syndrome. On the site http://healthtalk.org/peoples-experiences/long-term-conditions/alport-syndrome/overview, there are around 27 topic summaries with 250 video clips chosen to reflect issues important to participants, such as the first signs and symptoms of Alport syndrome, getting a diagnosis of Alport syndrome, medication, reproductive choices, living as a female ‘carrier’ and the emotional side of living with Alport syndrome. Although this study was UK based, many of the overall experiences are similar across the world—in practice, this means that when a person with Alport syndrome looks at any of these summaries, they should find that an experience or perspective akin to their own is included, although this may not necessarily be reported by a person of the same age, family situation or social class as themselves. This baseline understanding of UK patients is critical in order to inform further projects and toolkits to better support patients in the future, including improving communication, more effective and efficient clinical care, much needed mental health support and the treatment of female ‘carriers’.

## Summary

Overall, this review highlights the current and significant developments in Alport syndrome research from clinical and basic science to genetics and qualitative research with patients. The collaboration between clinicians, patients and researchers is key to accelerating the current research efforts in order to deliver the unmet needs of patients living with Alport syndrome.
